# Supplementary material for: Effects of Medium Cut-Off Polyarylethersulfone and Polyvinylpyrrolidone Blend Membrane Dialyzers in Hemodialysis Patients: A Systematic Review and Meta-Analysis of Randomized Controlled Trials
Source: Membranes (Basel). 2022 Apr 20;12(5):443. doi: 10.3390/membranes12050443 (PMC9144787; doi:10.3390/membranes12050443)

membranes

MDPI

Supplementary Material

Effects of Medium Cut-Off Polyarylethersulfone and Polyvinylpyrrolidone Blend Membrane Dialyzers in Hemodialysis Patients: A Systematic Review and Meta-Analysis of Randomized Controlled Trial

Yu-Hui Hung, Tai-Shuan Lai, Mohamed Belmouaz, Ya-Chun Tu, Chun-Fu Lai, Shuei-Liong Lin and Yung-Ming Chen

Supplementary Table S1. The MeSH search terms in the search strategy.

#	PubMed search terms
1	Expanded hemodialysis
2	HDx
3	medium cut-off membrane
4	medium cut-off polyvinylpyrrolidone blend membrane
5	MCO
6	high retention onset membrane
7	#1 OR #2 OR #3 OR #4 OR #5 OR #6
8	beta2-microglobulin
9	kappa free light chain
10	lambda free light chain
11	interleukin-6
12	albumin
13	#8 OR #9 OR #10 OR #11 OR #12
14	#7 AND 13
**#**	**EMBASE search terms**
1	('expanded hemodialysis' OR (expanded AND ('hemodialysis'/exp OR hemodialysis)) OR 'hdx'/exp OR hdx OR 'medium cut-off membrane' OR (('medium'/exp OR medium) AND 'cut off' AND ('membrane'/exp OR membrane)) OR 'medium cut-off polyvinylpyrrolidone blend membrane' OR (('medium'/exp OR medium) AND 'cut off' AND ('polyvinylpyrrolidone'/exp OR polyvinylpyrrolidone) AND blend AND ('membrane'/exp OR membrane)) OR mco OR 'high retention onset membrane' OR (high AND ('retention'/exp OR retention) AND onset AND ('membrane'/exp OR membrane)))
2	((('beta2 microglobulin' OR kappa) AND free AND light AND chain OR lambda) AND free AND light AND chain OR 'interleukin 6' OR albumin)
3	#1 AND #2
**#**	**Cochrane central registers of controlled trial databases**
1	(Expanded hemodialysis) OR (medium cut-off membrane) OR (high retention onset membrane) (Word variations have been searched)
2	(beta2-microglobulin) OR (kappa free light chain) OR (lambda free light chain) OR (interleukin-6) OR (albumin)
	#1 AND #2
	**ClinicalTrials.gov registry**
Status	All studies
Condition or disease	End-stage-renal disease
Other terms	medium cut-off polyvinylpyrrolidone blend membrane, medium cut-off membrane, MCO, expanded hemodialysis, HDx, high retention onset membrane, HRO

Supplementary Figure S1. Funnel plot of beta2-microglobulin studies. Right limb missing was found in the funnel plot, suggesting a potential publication bias.

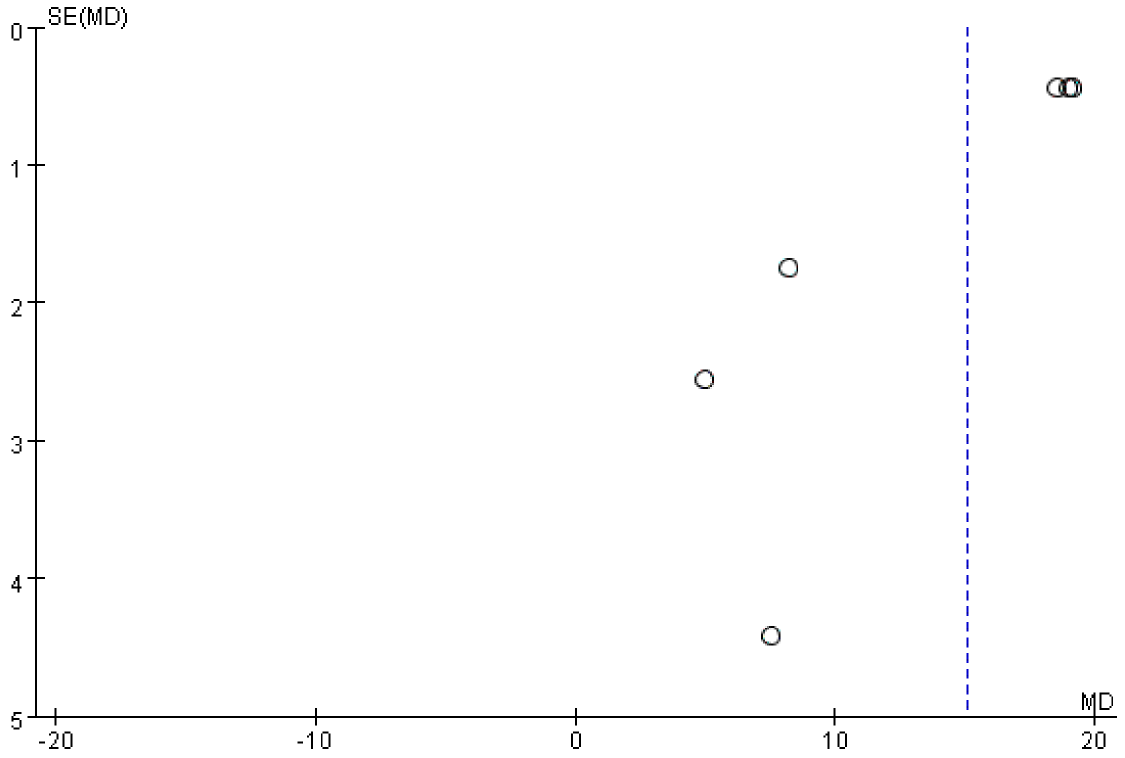

Supplement: Supplementary file 1 [file membranes-12-00443-s001.zip › membranes-1684744-supplementary.pdf]
